# Effect of time-dependent forcing on pole reversals in a conceptual dynamo model

**DOI:** 10.1038/s41598-026-48443-0

**Published:** 2026-04-24

**Authors:** Mátyás Herein, Lukács Kuslits, Dániel Jánosi

**Affiliations:** 1 HUN-REN Institute of Earth Physics and Space Science, Csatkai Endre utca 6-8, Sopron, H-9400 Hungary; 2 HUN-REN-ELTE Theoretical Physics Research Group, Pázmány Péter Sétány 1/A, Budapest, H-1117 Hungary; 3 Department of Theoretical Physics, Eötvös Loránd University, Pázmány Péter Sétány 1/A, Budapest, H-1117 Hungary

**Keywords:** Mathematics and computing, Physics

## Abstract

In this paper, we present a new, statistical framework for understanding the dynamics of polarity reversals under time-dependent forcing. The methods are thought to be generally applicable to geodynamo models in any scenario involving a time-dependent forcing. We demonstrate this framework on a simple conceptual dynamo model, the Rikitake system, which is a classical model of polarity reversals, known for its chaotic dynamics. Here, we investigate, for the first time, a non-autonomous extension of this model, in which the coupling parameter changes in time, mimicking the gradual activation or strengthening of a geodynamo. Given the non-autonomous nature of the underlying dynamics, the typical behavior of the system can only be accurately characterized using ensemble-based statistical approaches (instead of time series-based ones), in line with the so-called snapshot attractor view of dynamical systems theory. Using ensemble simulations with a “spin-up” initialization, we analyze the statistical behavior of polarity reversals, specifically, we construct the time-dependent ensemble-based cumulative distribution functions (CDFs) of chron lengths. Results show that as the system evolves and the coupling increases, longer chrons become increasingly permitted, albeit with lower probability. This behavior can be interpreted as the analogy of very rare “superchron” events, supported by paleomagnetic evidence. We find that early-stage chron distributions follow Gaussian statistics, while later stages exhibit lognormal distributions, indicating a transition in the underlying dynamical behavior as the system evolves. An intriguing and new result is the simultaneous probabilistic coexistence of a plethora of chrons with a multitude of lengths, whose ensemble characterizes the system at each point in time. These findings highlight the importance of incorporating ensemble methods in advanced geodynamo modeling.

## Introduction

The geodynamo, the mechanism responsible for generating Earth’s magnetic field, has been a topic of scientific investigation for over a century. At its core, the process involves the motion of electrically conductive fluids - primarily molten iron and nickel - within the Earth’s outer core. These fluids are subject to thermal and chemical convection^1,2^, the Coriolis force due to Earth’s rotation, and magnetic induction, which drive the generation of a self-sustaining magnetic field^3,4^.

This geomagnetic field plays a vital role in protecting life on Earth by acting as a shield against harmful solar and cosmic radiation, preventing the erosion of the atmosphere and maintaining conditions conducive to life^5,6^. Understanding the nature of the geodynamo is crucial for explaining the origin and dynamics of Earth’s magnetic field and gaining insights into the broader processes that govern planetary interiors and their evolution.

Theoretical models of Earth’s geodynamo have come a long way, thanks to results in fluid dynamics and computer simulations. Early models, like those based on the “dynamo equations” like the dynamos of Cowling^3^ and Elsasser^4^, were a big step in understanding how a rotating, convecting viscous fluid can create and keep a magnetic field. But these models often made things simpler, like assuming a steady state or a perfectly symmetrical shape, which could not fully capture the real complexity of Earth’s dynamo^7^. A breakthrough in geodynamo research came in the 1990 s when Roberts and Glatzmaier successfully simulated a fully self-sustaining geodynamo using a three-dimensional numerical model^8^. This achievement was a milestone in the field because it provided the first concrete evidence that the Earth’s magnetic field could indeed be generated and sustained by the complex interactions of convection, rotation, and magnetic induction.

Today, highly advanced direct numerical simulations are primarily used to create more realistic representations of the geodynamo process^9^. In addition, variational data assimilation applied to ensembles of slightly less complex dynamic models has shown promise in providing more reliable forecasts of short- to mid-term changes in the magnetic field^10^.

Researchers have also long sought simpler analogies that can still provide valuable insights into the basic nature of the geodynamo, see e.g. the works of Hetzenberg^11^ and Noziéres^12^, with new results appearing recently as well^13–15^. One of the most important of these is the two-disk dynamo model originally proposed by Rikitake^16^, which captures key qualitative features of the geomagnetic field, such as self-sustaining energy conversion (dynamo action) and the occurrence of pole reversals.

Despite the availability of advanced three-dimensional geodynamo simulations, several key questions remain unresolved. One such question is about the exact physical reasons behind the occurrence and the traits of pole reversals observable in the paleomagnetic record. A number of recent studies aimed at exploring the parameter space and features of the increasingly large set of available simulations searching for the conditions prone to generate the kind of reversing models which can also be considered earth-like in every other aspect^17–19^. However, a major problem remains that no comprehensive, representative reversal statistics is currently available for simulations otherwise considered earth-like, as it would require prohibitive computational time and capacity^19^.

Another issue concerns the time evolution of the geomagnetic field, specifically how the magnetic field develops from the initial field required to initiate the dynamo process. This transition is one which is inherently non-autonomous, that is, during the process some parameters describing the system (e.g. the Rayleigh or Elsasser numbers) can become time-dependent, while these parameters are usually treated as constants in today’s advanced geodynamo simulations^20,21^. At the same time, recent paleomagnetic evidence points in the direction of such a “switching on” of a strong Hadean geodynamo, as the authors of Tarduno et.al^22^. find that the strength of the total magnetic field might have increased by a factor of two during the Hadean and Archean eons (approx. 4.2-3.2 Ga). Furthermore, it was reported in LeBars et.al^23^., using an experimental setup, that during the “early” stages of the dynamo, there were brief episodes of magnetic field bursts. After these short bursts, longer phases were observed where the magnetic fields were steady and gradually increasing, signaling the onset of a dynamo.

This paper aims to address these issues from the point of view of the basic phenomenon of reversals and reversal statistics in a simple chaotic dynamo model. We study the Rikitake system as a simple model analogous to the geodynamo, for which robust reversal statistics can be produced. We modify it to include an explicitly time-dependent coupling term that also serves as a forcing term, mimicking the onset and strengthening of the geodynamo through long-term variations affecting the thermal convection in the Earth’s outer core. We apply the knowledge accumulated in dynamical systems theory regarding non-autonomous (explicitly time-dependent) systems, namely, that following only one trajectory cannot be statistically representative, and a clear picture can only be obtained if we follow an ensemble of trajectories. Statistical analysis then has to be performed over this ensemble in each time instant, rather than over an individual time series. This way, the probability distribution of chrons, i.e. the times between reversals, itself becomes time-dependent. We study its time evolution, and find that as time goes on, longer chrons become permitted (with limited probability). As a new feature of this method, these long chrons coexist, in a probabilistic sense, with shorter ones, with their materialization being determined by the probability distribution.

## The Rikitake system

### The classical, autonomous case

While the basic framework for understanding dynamo action had already long been established by the 1960 s, the complex and chaotic nature of Earth’s magnetic field proved to be harder to capture. This is where the work of Rikitake proved to be crucial^16,24^. The Rikitake model demonstrated how dynamo action could lead to sensitive dependence on initial conditions, and it was one of the first to show the irregular, chaotic behavior of Earth’s magnetic field. The chaotic dynamics of Rikitake’s model anticipated what we now know to be true: the geodynamo is inherently nonlinear, characterized by fluctuations and reversals, rather than operating in a simple, steady-state mode^25,26^. These insights into chaotic behavior, increasingly recognized in Earth’s magnetic field, set the stage for later advancements in numerical simulations and theoretical models^27,28^.

Various studies have examined the interpretability of the Rikitake model and its relevance to the geodynamo^29^. It has to be noted, that some criticism has been raised too due to its perceived inability to reproduce an Earth-like distribution of geomagnetic pole reversals, as seen in paleomagnetic records^30^. Despite this, over the years, the Rikitake system has become a solid basis for understanding chaotic dynamo behavior, due to its ability to reproduce key features of magnetic field generation, such as reversals, variations in field strength, and irregular cycles. As a result, it has been applied not only to understanding Earth’s magnetic field but also to studying other planetary and stellar dynamos, where similar chaotic behaviors are observed^31^.

The Rikitake system is derived from a simplified two-disk dynamo apparatus, exhibiting a rich and complex dynamical behavior, including the presence of a Lorenz-type chaotic attractor around its two singular points^16^. The global dynamics of the model have been extensively studied, revealing a variety of phenomena such as the creation of the chaotic attractor, the existence of invariant planes with completely integrable dynamics, and the presence of orbits that diverge towards infinity^30,32^. The system is made up of the three coupled first-order differential equations

1 $$\begin{aligned} \dot{x}&= -bx + yz \nonumber \\ \dot{y}&= -by - ax + xz \nonumber \\ \dot{z}&= 1 - xy, \end{aligned}$$

where *x* and *y* are related to the electric currents induced in the disks, *z* is related to the angular velocities of the disks, *a* is the coupling and forcing term and *b* is the dissipation strength^33^.

**Fig. 1:**
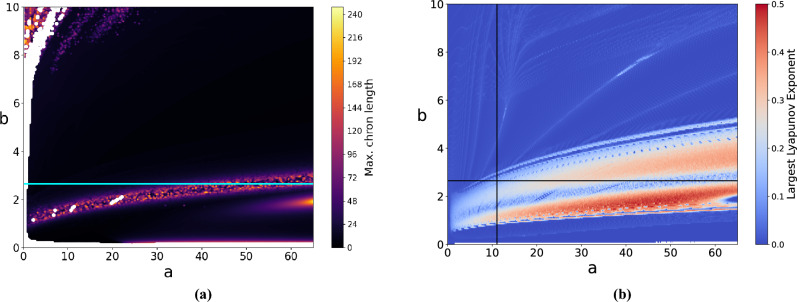
Exploring the parameter space. (**a**) The maximum chron length. The light blue line indicates $b = 2.65$, a value going far into the band with distinctly larger chrons. The white regions mean parameter values where the system does not produce reversals. (**b**) The largest Lyapunov exponent. The horizontal line is at $b = 2.65$, and the vertical one is at $a = 11.05$, the start of the chaotic regime along the horizontal line.

The system exhibits different behaviors depending on the values of these parameters. Figure 1 reveals the parameter landscape in two key features. Namely, we show the maximal length of chrons (i.e. the time between two reversals), being of particular interest to our analysis (panel a), and the largest Lyapunov exponent (panel b). The former reveals a distinct band in which chron lengths are significantly larger - and more variable - than in other regions, while the white dots indicate parameter pairs where no reversals occur. As it will become clear later, our method is best demonstrated when exploring regimes with different chron lengths, thus we choose the value $b = 2.65$ (light blue line) for all subsequent simulations, where a great variability of chron lengths is found. Figure 1ais supplemented with a similar one in the Supplementary Material, showing the number of chrons within a time series, where the band of long chrons found here translate into a band of smaller number of chrons.

The Lyapunov landscape of Fig.1breveals parameter values with chaotic behavior. For the chosen $b = 2.65$ dissipation strength (horizontal black line), the system is mostly inside the chaotic regime, apart from the range $a \lesssim 10$, while for larger *a* values ($\gtrsim 45$) the pattern of the Lyapunov exponents is irregular. This is consistent with the emergence of periodic windows in an otherwise chaotic regime, which we demonstrate by showing the bifurcation diagram along the $b = 2.65$ line in the Supplementary Material. The vertical black line belongs to $a = 11.05$, a value which we determine to be safely inside the chaotic regime along the horizontal line.

**Fig. 2:**
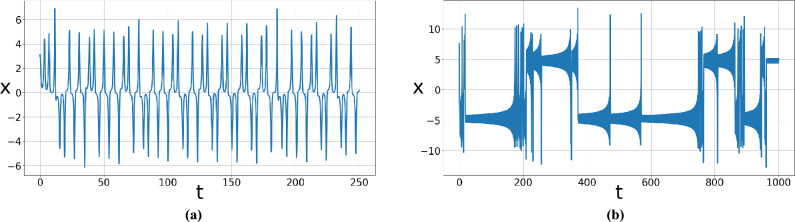
Time series in the *x* variable of system (1), with $b = 2.65$, while $a = 11.05$ (a) and $a = 60$ (b). In both cases the dynamics is chaotic and reversals are present, but, as evident from the figure, the chrons are quite short in the first case, while longer ones can be observed in the second case.

Different chron lengths are demonstrated in Fig.2, where we show the time series in the *x* variable for $a = 11.05$ (panel a) where the time between reversals is very short, and $a = 60$ (panel b), where longer chrons can be observed along short ones. In the Rikitake model, we find that reversals can be best observed in the *x* variable, thus we will use this for further analysis in all following cases.

### The non-autonomous case

Here we introduce the non-autonomous Rikitake model, wherein one of the system’s parameters is made explicitly time-dependent to capture the onset of the time-dependent geodynamo. We achieve this by writing the coupling term as

2 $$\begin{aligned} a(t) = a_0 + \nu t. \end{aligned}$$

The new parameter introduced is the *rate* of the parameter change, and we choose it to be $\nu = 0.0325$. Here $a_0 = 11.05$ represents the initial state of the system. This choice is based on the fact that at this value the $\nu = 0$ autonomous system is already chaotic while also exhibiting reversals, as Figs.1b and 2a show, i.e. this can be regarded as a base state from which the “switching on” can take place.

There is a vast amount of literature covering the dynamics of chaotic non-autonomous (i.e. explicitly time-dependent) dynamical systems, similar to the one defined above, see e.g. Romeiras et.al^34^., as well as others^35–39^, for a recent review see Jánosi and Tél^40^. Their most important property is that following only one time series in them is not statistically representative. This is because, while for autonomous systems the shape of the chaotic attractor is constant, in non-autonomous systems this shape is time-dependent, becoming a so-called snapshot attractor^34^. For autonomous chaotic attractors, following a single trajectory for a long (formally infinite) time is sufficient to characterize the attractor, since eventually all of its parts will be visited and all possible states will be explored. At the same time, following an ensemble of trajectories (again, formally infinitely many) for a much shorter time also results in tracing out the shape of the attractor, as different trajectories end up in different parts of it. Therefore, autonomous chaotic attractors have the ergodic property^41,42^.

By contrast, snapshot attractors cannot be traced out by a single trajectory (even if it is infinitely long), since at every instant it only represents one state of the extended attractor (with many permitted states) belonging to that instant only^39^. Thus it does not make sense to follow one trajectory to infinity because it would only provide information about one state of the snapshot attractor. However, following an ensemble of trajectories is effective here as well, since the large number of trajectories can explore all permitted states at every instant. Thus, in non-autonomous systems exhibiting chaos, ergodicity is lost, in the sense that long-time averages are no longer equivalent to ensemble averages^34,43^, and the ensemble-based method is superior to the single-trajectory one.

It is important to note that the most well-known application of the theory of non-autonomous systems is the effort in understanding climate change. The Earth’s climate is a complicated turbulent system exhibiting chaotic behavior. The monotonic change of a parameter, e.g. the $\hbox {CO}_2$ concentration, results in a non-autonomous chaotic system. There are numerous publications dedicated to this aspect of the climate, for reviews see e.g. Dijkstra^44^ and others^45–47^. In the context of climate, a trajectory ensemble was termed “parallel climate realizations”, introduced by Herein et.al^48^. (for subsequent papers see^49–51^), an interpretive picture asserting that there exists a large number of possible states (realizations) of the climate at the same time (parallel), but of course only one materializes, the one that can actually be measured. For dynamical systems, the similar picture of “parallel dynamical evolutions” was introduced in Jánosi et.al^52^..

Another important aspect of climate worth taking over to dynamical systems theory is the concept of convergence times. Essentially, it takes some time until a trajectory ensemble fully spreads over the snapshot attractor, leaving the beginning of the simulation non-evaluable^46,51^. To combat this, it is common practice to simulate the autonomous system with the initial parameter value, for a sufficiently long time for the trajectories to spread on the (yet stationary) attractor, and then switch on the drift. This so-called “spin-up” phase is typically not very long for dynamical systems, but can be long for the climate, especially for the oceans^53^.

Regarding the non-autonomous Rikitake system in the present paper, we are taking over the method of trajectory ensembles and parallel dynamical evolutions, simulating 100000 trajectories. We apply the “spin-up” method in order to avoid dealing with convergence to the snapshot attractor while the parameter change takes place. The trajectories are initially randomly distributed in the phase space around the point (1, 3, 1) with an average radius of $10^{-9}$. These trajectories are integrated in the autonomous system with $a = a_0 = 11.05$ for the sufficient spin-up time $t_0 = 1000$, which is rather long for a simple dynamical system. The duration of the spin-up is decided based on how the autocorrelation, as well as the difference between the means of two differentially initialized ensembles, converge to a value that is numerically indistinguishable from zero, meaning that by the end of this phase trajectories have most certainly spread over the attractor, and all states are thought to be properly represented^54^. The result of this process can also be regarded as a “biased” dynamo, with some preliminary value of the parameter, here the coupling *a*. Then, the parameter drift is turned on with $\nu = 0.0325$. The initial conditions for the non-autonomous system are the end states of the autonomous simulation. The non-autonomous system is observed until $t = 1660$ (after the spin-up), during which the coupling increases to $a_{max} = 65$. Note that after this value we would leave the band of longer chrons on the autonomous parameter landscape of Fig.1a.

## Ensemble statistics of chrons

Earth’s magnetic field, while having a complicated time series, has the tendency to keep the sign of its polarity constant for a considerable amount of time. Such intervals, i.e. the waiting times between reversals in the observed (or simulated) magnetic field are called chrons^55^. Chronologically, their durations are highly variable, ranging from several tens of thousands of years (kyr) to several tens of millions of years (Myr), including extended intervals known as superchrons - such as the Cretaceous Normal Superchron, which persisted for approximately 40 Myr^56^. One of the ways the understanding of the geodynamo has been attempted is through the distribution of the length of chrons. A number of studies have been dedicated to this matter, with differing conclusions. There are suggestions of exponential (indicating a Poisson process)^57,58^, lognormal^59^, power-law^60^, gamma^61,62^ and Lévy^63^ distributions for the observed chrons. Regarding simple dynamo systems, in Gissinger^14^ an exponential distribution was found. It is worth noting that an exponential distribution has also been observed in the autonomous Rikitake system^58,64^.

Here we are conducting a similar analysis in the non-autonomous Rikitake system. A naive approach would be to simulate a long trajectory and study the statistics of its chrons - however this, as we stated before, is not a representative analysis. One must note that this is precisely what is happening when studying the observed magnetic field as well: the statistics is made based on a single time series. For this reason, we assert that, in case of a parameter drift, this type of investigation cannot be sufficient, as the statistics itself can drastically change in time, as we shall demonstrate.

We use the methods described in the previous section, simulating 100000 trajectories and using time-dependent ensemble statistics (instead of temporal ones for each individual trajectory). That is, we ask how long of a chron can be observed for each trajectory at given time instants. In order to make our analysis easier, we concentrate on the moments of the reversals themselves, and look at the lengths of the chrons preceding them in each trajectory. We then construct the cumulative distribution function (CDF) of the chron lengths belonging to the given reversal, across all trajectories, i.e. the ensemble. We then repeat this process for the sequence of reversals in the simulation. The reversals are identified based on the time series of variable *x* in all trajectories.

**Fig. 3:**
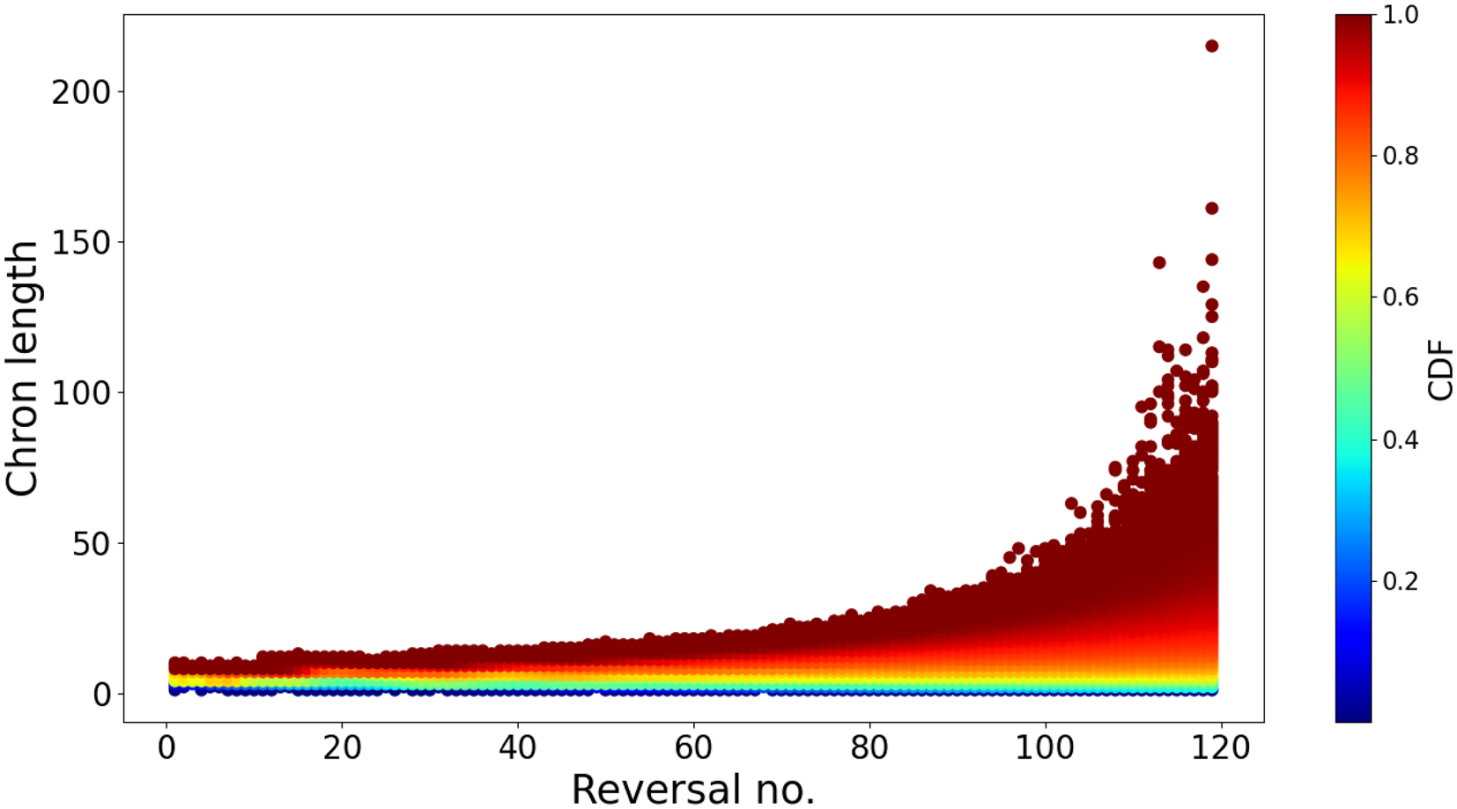
The permitted lengths of chrons in the ensemble, given at the observed number of reversals in each trajectory, and the CDFs of the chron lengths. The CDFs are calculated individually for each reversal, thus each “column” in the image has a separate CDF.

Figure 3 shows the sequence of reversals and the corresponding chron lengths detected, with their CDF shown as a colormap. Note that the *n*th reversal in one trajectory typically does not happen at the same time as the *n*th reversal in another trajectory, since the chron lengths in the two trajectories might be significantly different. That is, the horizontal axis of Fig.3 is not ordered in time, but according to the reversals. The first observation is that as time goes on (the more reversals are observed), i.e. as the coupling parameter increases, longer and longer chrons are detected. In the beginning, the maximum chron length barely reaches 10, while by the last reversal (number 119) it surpasses 200. From the CDF, it can be inferred that the probability of the appearance of these long chrons in an ensemble is quite low, as the CDF value for them is close to 1. However, it is indeed notable that they show up at all, since one of the drawbacks of the Rikitake model was thought to be the lack of such long chrons^13^.

**Fig. 4:**
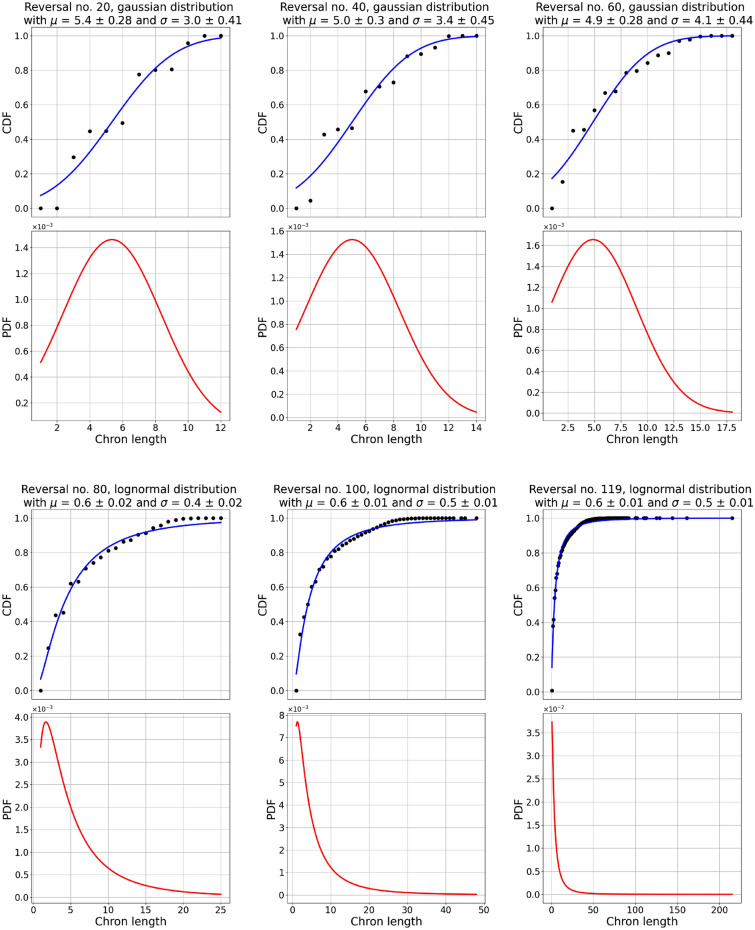
The chron lengths of select reversals from Fig.3 (black dots), with the fitted CDFs (blue curves) indicated in the panel titles. The corresponding PDFs (red) are the numerical derivatives of the blue curves.

For more detailed understanding, we selected some reversals from Fig.3, showing the calculated CDFs in Fig.4. What we see is that in the beginning of the simulation (the left side of Fig.3), a Gaussian CDF of the form^1^

3 $$\begin{aligned} \Phi \bigg (\frac{x - \mu }{\sigma }\bigg ) = \frac{1}{\sqrt{2\pi }}\int ^{\frac{x - \mu }{\sigma }}_{-\infty }{e^{-u^2/2}du} \end{aligned}$$

can be fitted (blue line) to the simulated chron lengths (black dots), where $\mu$ and $\sigma ^2$ are the expectation value and variance of the distribution. The probability density function (PDF) is then calculated as the numerical derivative of the fitted curve, and is plotted in red under the CDF. The 20th, 40th and 60th reversals, displayed in the upper row, are fitted with the distribution above. A trend can be observed for these images: while the center of the distribution, $\mu$, remains roughly the same, more low-probability regions can be observed on the right, as longer chrons become permitted at later reversals, as a consequence of the increase in the parameter *a*.

In the later stages of the simulation, where the value of the parameter has increased more significantly, instead of Gaussian, a lognormal CDF can be fitted to the chron lengths. This takes the form

4 $$\begin{aligned} \Phi \bigg (\frac{\ln {x} - \mu }{\sigma }\bigg ), \end{aligned}$$

where $\Phi$ represents the same function as in (3) and $\ln ()$ indicates the natural logarithm. The 80th, 100th and 119th reversals (lower row) are fitted with this distribution. As longer chrons become permitted, the distribution becomes more skewed (since again the maximum does not move on the horizontal axis), until at the 119th reversal it becomes effectively one-sided. This again illustrates the fact that with larger values of *a*, longer chrons can be observed, but with small probability at a given reversal. As for hypothesis testing, the Supplementary Material contains q-q plots for each case presented here, confirming the validity of the fitted distributions.

## Conclusions

Our work introduced a new framework for addressing time-dependence in geodynamo modeling. The framework is mathematically well-founded, being based on the concept of snapshot attractors. As a simple example, we explored the chaotic dynamics of the non-autonomous Rikitake dynamo system, focusing on the statistics of polarity reversals. Owing to its low dimensionality, this simple model provides an efficient and practical environment for exploring new ideas via statistically robust large ensembles, an approach that would be computationally far more challenging in complex dynamo models. By introducing a time-dependent driving term, we mimicked the gradual “switching-on” and strengthening of a geodynamo. Using ensemble-based methods with a set of trajectories, we demonstrated that the non-autonomous system fundamentally differs from the autonomous one, where the permitted chron lengths would not change considerably throughout the simulation^24,58^. Our results revealed a time-dependent probability distribution of chrons, showing that longer intervals between reversals can become permitted as the dynamo evolves. This naturally means that, statistically, many chron lengths are allowed at a given time, but only one “manifests” in a single time series. Based on the CDF of reversals, it is clear that the probabilities of longer chrons are significantly lower than those of shorter chrons. We note that, speaking strictly in terms of analogies, similar behavior is observed in paleomagnetic records, where “superchrons” are rare events^65^.

However, we emphasize that the Rikitake system is a highly oversimplified dynamo model, thus these results cannot be compared to, and are not meant to give any conclusion about, the paleomagnetic observations. It has long been a question whether the Rikitake system possesses any direct connection to the magnetohydrodynamic (MHD) equations. As it has already been pointed out by Moffatt^7^, this is a very challenging task, with no guarantee of a closed-form solution. Such a formulation, if the derivation can be rigorously established, could provide physically consistent scaling for the relevant variables and may offer a basis for interpreting the Rikitake system in a geophysical context. Nevertheless, even in this case, the Rikitake system would only remain a dynamical analogue of a true MHD geodynamo.

Based on the calculated time-dependent PDF, we found that the initial (weaker dynamo) state follows a Gaussian distribution, while the stronger state follows a log-normal distribution. These findings may align with existing literature supporting an emergence of high variability and time-varying tendencies in chron lengths produced by simulations associated with non-autonomous “external” influences on the system, such as a steadily cooling inner core, tidal deceleration or a change in core-mantle boundary heat flux^66–69^, although our simulation is very simple compared to these results. Our results also challenge prior assumptions about the Rikitake model’s inability to reproduce Earth-like chron distributions^30^.

These findings highlight the importance of including non-autonomous effects or time-dependent parameters in geodynamo simulations, and point to the conclusion that capturing the dynamics of any transitional phase and the chaotic magnetic reversals in such a regime is only possible within the ensemble framework. The long-term evolution of the current geodynamo is intrinsically governed by the thermal history of the core^2^, necessitating the investigation of time-dependent forcings in geodynamo models. Incorporating time-dependence in boundary conditions or non-dimensional parameters might be necessary for establishing simulations which realistically represent the long-term evolution and initiation of the geodynamo along with an Earth-like distribution of polarity reversals. While there has been many efforts exploring a long-term time evolution either via time-dependent forcing directly introduced to models^66^, or (to some extent) indirectly through sequences of models with varying parametrization^70,71^, no such attempt relying on ensemble statistics is known to the authors. At the same time, ensemble approaches are already used in examining the present, stationary geodynamo without any time-dependent parameters introduced, see e.g. Fournier et.al^72^.. Combining these techniques could further improve the fidelity of dynamo models, offering a robust framework for studying planetary and stellar magnetic fields.

### Supplementary material

The Supplementary Material contains the following figures: a parameter landscape showcasing the number of chrons in a time series in the autonomous system, the bifurcation diagram for $b = 2.65$, and q-q plots confirming the validity of the distributions in Fig.4.

## Supplementary Information

Supplementary Information.

## Data Availability

The data that support the findings of this study are available from the corresponding author upon reasonable request.

## Footnotes

1 For the numerical treatment of the distributions, the *scipy.special* Python library was used.
