# Supplementary material for: Effect of time-dependent forcing on pole reversals in a conceptual dynamo model
Source: Sci Rep. 2026 Apr 24;16:18981. doi: 10.1038/s41598-026-48443-0 (PMC13276039; doi:10.1038/s41598-026-48443-0)
Supplement: Supplementary file 1 — Supplementary Information. [file 41598_2026_48443_MOESM1_ESM.pdf]

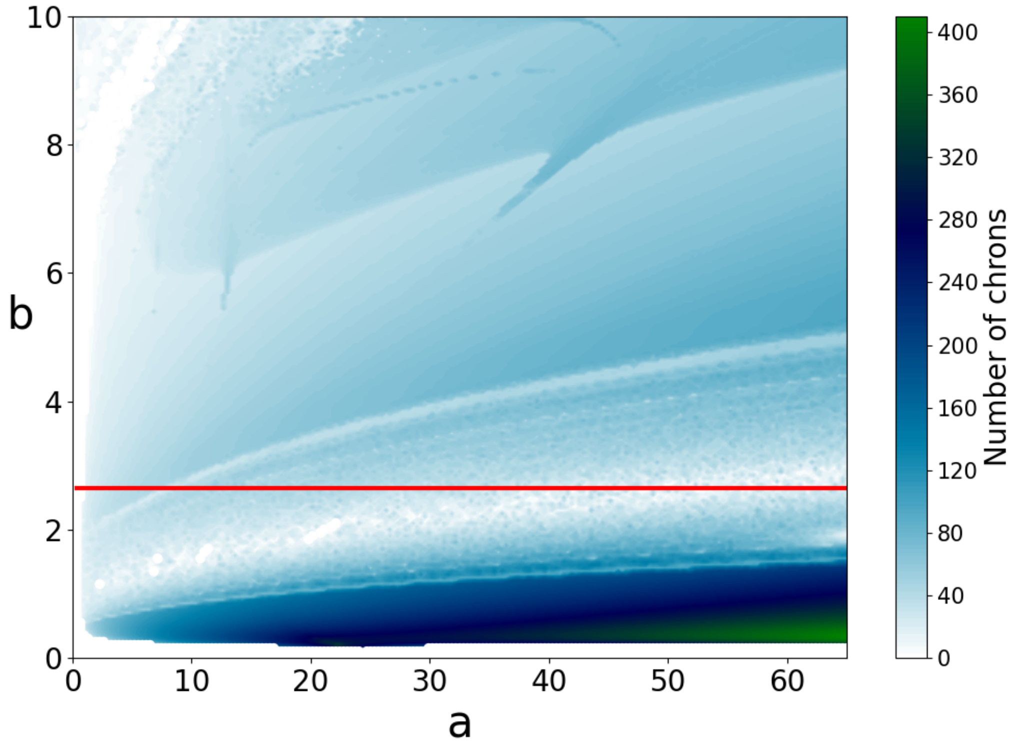

Figure S1: Parameter landscape with the number of chrons within a time series shown as colormap. The band of small chron numbers (light colors) matches the band of long chrons in Fig.1a, with the $b = 2.65$ red line inside this band.

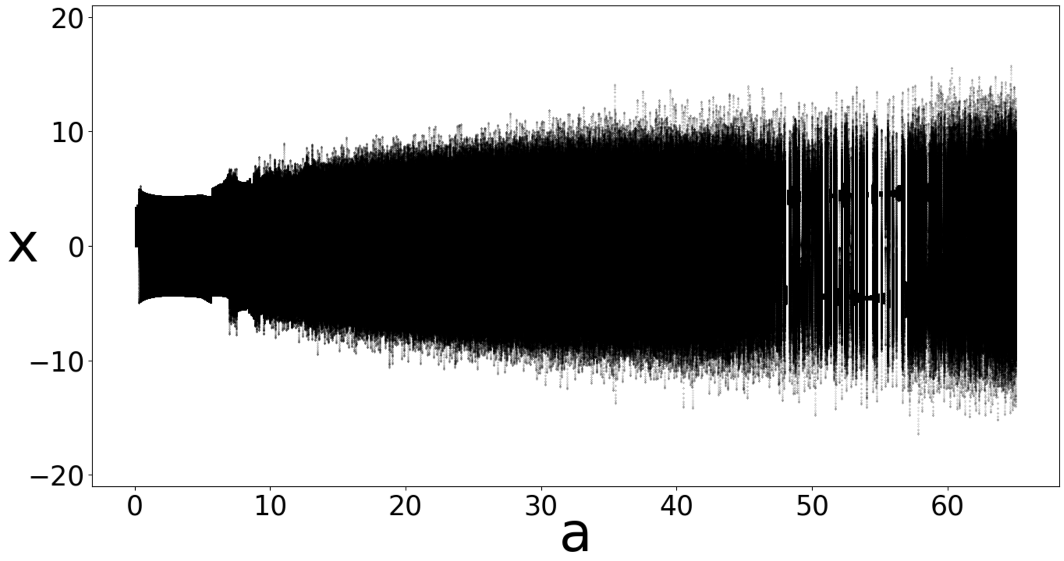

Figure S2: Bifurcation diagram for $b = 2.65$. Because of the reversals, there are no narrow sections even in the non-chaotic regime. Rather, regularity is represented as the diagram having smooth edges for small a values, while irregular edges for $a \gtrsim 10$ signal chaos. Between values of around 45 and 55, periodic windows can be observed, which correspond to the irregular Lyapunov exponents found along the horizontal line in Fig.1b.

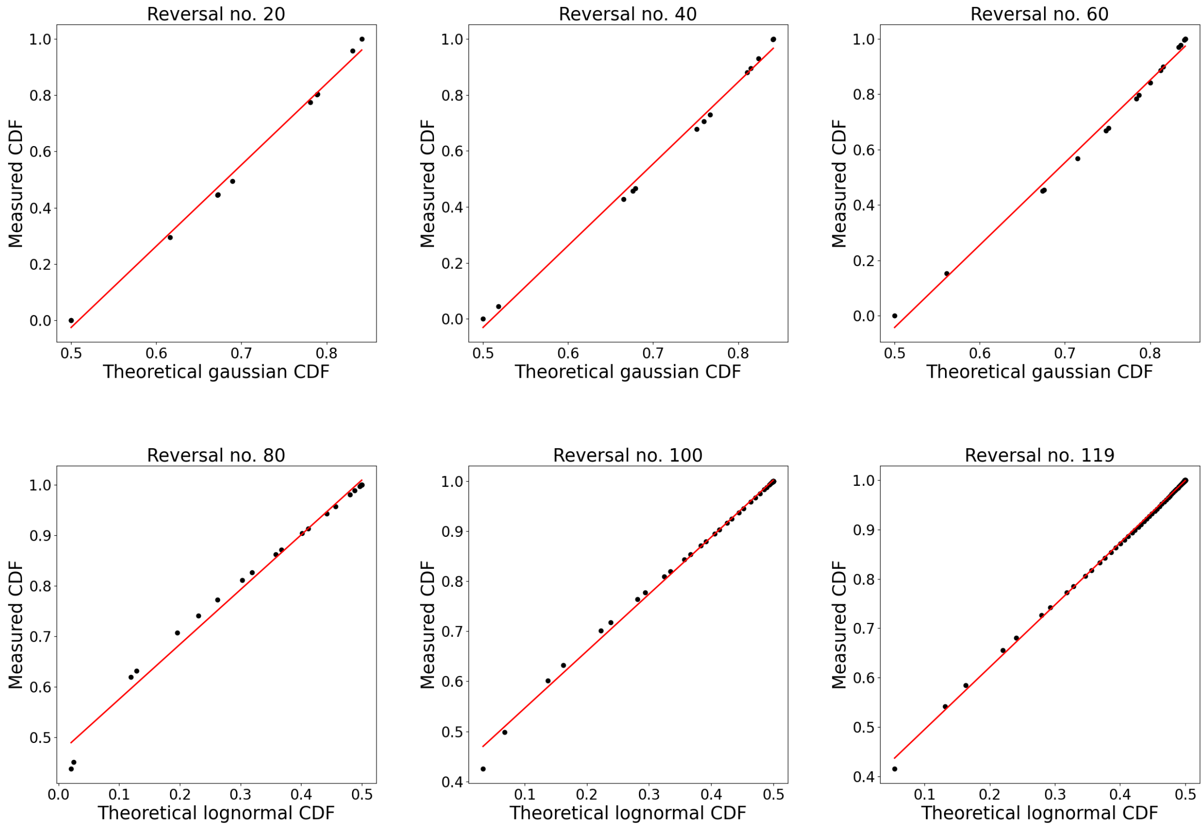

Figure S3: The q-q plots for the CDFs presented in the main text. In all cases, the straight line fits prove the validity of the distributions.
